# Elevational Differences in Plant Growth Traits, Litter Decomposition, and Soil Bacterial Communities During *Deyeuxia angustifolia* Encroachment in Alpine Tundra

**DOI:** 10.3390/plants15182776

**Published:** 2026-09-10

**Authors:** Yueming Zhao, Jian You, Wei Zhao, Yulong Li, Yujiao Zhang, Ming Xing, Alimu Wubuli, Xia Chen

**Affiliations:** 1 National & Local United Engineering Laboratory for Chinese Herbal Medicine Breeding and Cultivation, School of Life Sciences, Jilin University, Changchun 130012, China; zhaoyueming@cbom.bio (Y.Z.); jianyou@jlu.edu.cn (J.Y.); cbs1981@163.com (W.Z.); 15575119483@163.com (Y.L.); zyj2011905@126.com (Y.Z.); me9894@163.com (M.X.); 13201327012@163.com (A.W.); 2 Changbaishan Nature Conservation Management Center (Changbaishan Academy of Science), Yanbian 133613, China; 3 Yanbian Korean Autonomous Prefecture Academy of Agricultural Sciences, Yanbian 133002, China

**Keywords:** *Deyeuxia angustifolia*, plant encroachment, alpine tundra, growth trait variation, soil bacterial community, Changbaishan

## Abstract

Graminoid encroachment in alpine tundra is often reduced to a simple rise in dominant-species cover, leaving open whether this aboveground shift is coupled to belowground soil conditioning. Here, we tracked the encroachment front of *Deyeuxia angustifolia* (Kom.) Y.L.Chang in the Changbaishan alpine tundra, integrating plant community surveys, growth trait monitoring, litter decomposition, phenology-matched soil physicochemical properties, enzyme activities, and bacterial 16S rRNA sequencing across slightly and largely encroached plots at 2000 and 2200 m. Abundance-weighted plant community composition differed significantly across habitats (*p* = 0.0003), with *D. angustifolia* relative cover surging from 21.8–29.9% in S to 89.9–93.0% in L, substantially displacing *Rhododendron aureum* and reducing community Shannon diversity and Pielou evenness. Repeated-measures mixed models revealed that *D. angustifolia* exhibited elevation-dependent growth trait variation (elevation × stage × date interactions for height, leaf width, stem diameter, leaf count, and tillers; all *p* < 0.05): plants at 2000 m produced more tillers with a higher seasonal maximum tiller count (active and seasonal maximum tillers), whereas plants at 2200 m prioritized culm reinforcement and foliar expansion (increased leaf width, length, and stem diameter) alongside reduced leaf and branch numbers. Litter decomposition linked these patterns to belowground change: rates were similar between stages at 2000 m, but at 2200 m largely encroached plots decomposed more slowly (lower k, *p* < 0.001; higher September mass remaining, *p* = 0.002) while inorganic nitrogen and enzyme activities rose, indicating active local nutrient transformation despite slower turnover. Bacterial community composition varied mainly with elevation (*p* = 0.001), with further contributions from encroachment stage and month, reflecting compositional restructuring rather than simple diversity change. Together, these results depict *D. angustifolia* encroachment as a continuous process, from establishment through local space occupancy to litter decomposition dynamics and associated soil habitat differentiation, that reorganizes both aboveground structure and belowground habitats and offers ecological evidence for forecasting tundra meadowization under continued warming.

## 1. Introduction

Alpine tundra ecosystems are highly sensitive to climate warming because their vegetation structure, soil nutrient turnover and microbial processes are strongly constrained by low temperature and short growing seasons [1,2,3]. Under ongoing global warming, many alpine ecosystems are undergoing directional vegetation shifts, including the expansion of shrubs and herbaceous plants into habitats that were previously limited by low temperature [4,5,6,7]. Plant encroachment can alter not only aboveground community composition, but also litter inputs, soil microenvironment, nutrient cycling and microbial community structure, with potential consequences for ecosystem carbon and nitrogen dynamics [8,9,10]. Clarifying the coordinated changes between aboveground vegetation and belowground ecological processes is therefore essential for predicting the functional trajectory of alpine tundra ecosystems under environmental change [11,12,13].

The alpine tundra of Changbaishan is one of the few tundra ecosystems distributed in the mid- to low-latitude belt and represents one of the three major tundra regions in China [14,15,16]. In recent decades, this ecosystem has been increasingly affected by the upward expansion of *Deyeuxia angustifolia* (Kom.) Y.L.Chang [10,17]. As a graminoid species capable of both sexual reproduction and clonal propagation, *D. angustifolia* can form dense patches with high cover and gradually replace or suppress native tundra shrubs [18,19,20,21]. Its expansion may promote a shift from shrub-dominated tundra toward meadow-like vegetation [22]. However, the ecological mechanisms that enable *D. angustifolia* to persist and become locally dominant at the expansion front remain insufficiently understood. Whether its expansion is accompanied by coordinated changes in plant growth traits, soil nutrient conditions and soil bacterial community structure requires further investigation.

Growth trait variation is an important strategy by which plants adapt to harsh and heterogeneous environments [23,24]. For *D. angustifolia*, comparisons of litter decomposition between slightly and largely encroached habitats can indicate whether differences in local dominance are associated with variation in organic matter turnover [25]. At a plant encroachment front, these traits may respond differently to expansion intensity and elevation [26,27,28]. For example, relatively warm lower-elevation habitats may favor vertical growth and clonal propagation, whereas higher-elevation habitats may require adjustments in leaf and stem traits to cope with colder and more resource-limited conditions [29]. Therefore, examining the variation in growth traits of *D. angustifolia* across elevation and expansion stages can provide insight into its growth and reproductive strategies in alpine tundra ecosystems.

Litter decomposition provides an important process-level link between aboveground plant dominance and belowground habitat differentiation [30,31,32]. Changes in dominant plant species can modify the quantity and quality of litter entering the soil, thereby influencing decomposition rates, nutrient release and microbial activity [33,34]. Such as *D. angustifolia*, its litter decomposition in slightly and largely encroached habitats may indicate whether local expansion is associated with altered organic matter turnover [27,35,36]. Decomposition patterns between lower and higher elevations can help reveal whether the soil habitat context of the expansion front is linked to facilitation or constraint of litter decomposition dynamics and associated soil nutrient cycling.

Belowground soil habitat conditions may also be closely associated with the growth traits of encroaching plants [37,38]. Soil pH, organic carbon, total nitrogen, inorganic nitrogen and soil enzyme activities are closely related to nutrient availability and organic matter transformation [39,40]. Soil bacterial communities play important roles in litter decomposition, nitrogen transformation and nutrient cycling, and changes in their diversity and composition may indicate shifts in soil habitat status along plant expansion gradients [11,12,13]. However, previous studies on alpine plant encroachment have often focused separately on aboveground vegetation patterns or belowground microbial responses. Few studies have integrated plant community structure, growth trait variation, soil physicochemical properties, enzyme activities and bacterial 16S rRNA sequencing data within a unified habitat-level framework.

In addition, plant traits and soil microbial properties often exhibit strong seasonal dynamics [41,42,43,44,45]. In alpine ecosystems, the short growing season may lead to pronounced temporal variation in plant growth, nutrient demand and microbial activity [46,47,48]. Linking plant phenological observations with soil measurements from corresponding growing season months can help determine whether variation in plant traits is associated with soil nutrient status and microbial habitat differentiation [29,45,49]. Such an approach is particularly useful for evaluating above–below ground correspondence at the habitat level, while avoiding over-interpretation of direct causality [12,37,50,51].

In this study, we investigated the encroachment front of *D. angustifolia* in the alpine tundra of Changbaishan [15,22,28,52]. Plant community surveys, phenological monitoring of *D. angustifolia* growth traits, litter decomposition data, phenology-matched soil physicochemical properties, enzyme activities and bacterial 16S rRNA sequencing data were integrated across slightly and largely expanded plots at 2000 m and 2200 m. Specifically, we aimed to (1) determine whether plant community structure differs among elevation and expansion-stage habitats; (2) evaluate the growth trait variation of *D. angustifolia* at the expansion front; (3) assess whether decomposition of *D. angustifolia* litter differs between slightly and largely encroached habitats; and (4) examine habitat-level associations among plant growth traits, litter decomposition, soil properties, enzyme activities and bacterial community characteristics. This study provides an integrated perspective on aboveground trait adjustment and belowground habitat differentiation during alpine tundra plant encroachment.

While previous investigations in the Changbaishan tundra have documented broad-scale vegetation changes and snapshot soil properties associated with *D. angustifolia* expansion, two critical issues remain unaddressed: (1) how in situ litter decomposition kinetics vary across contrasting elevational encroachment fronts and (2) how aboveground vegetative growth phenology temporally aligns with belowground nutrient availability and microbial community turnover. Here, we integrate multi-interval phenological monitoring, in situ litterbag decomposition, soil enzymatic assays, and bacterial 16S rRNA sequencing across lower (2000 m) and higher (2200 m) encroachment zones to provide a temporally matched, habitat-level assessment of aboveground–belowground differentiation.

## 2. Results

### 2.1. Plant Community Differentiation Along the D. angustifolia Encroachment Gradient

**Figure 1:**
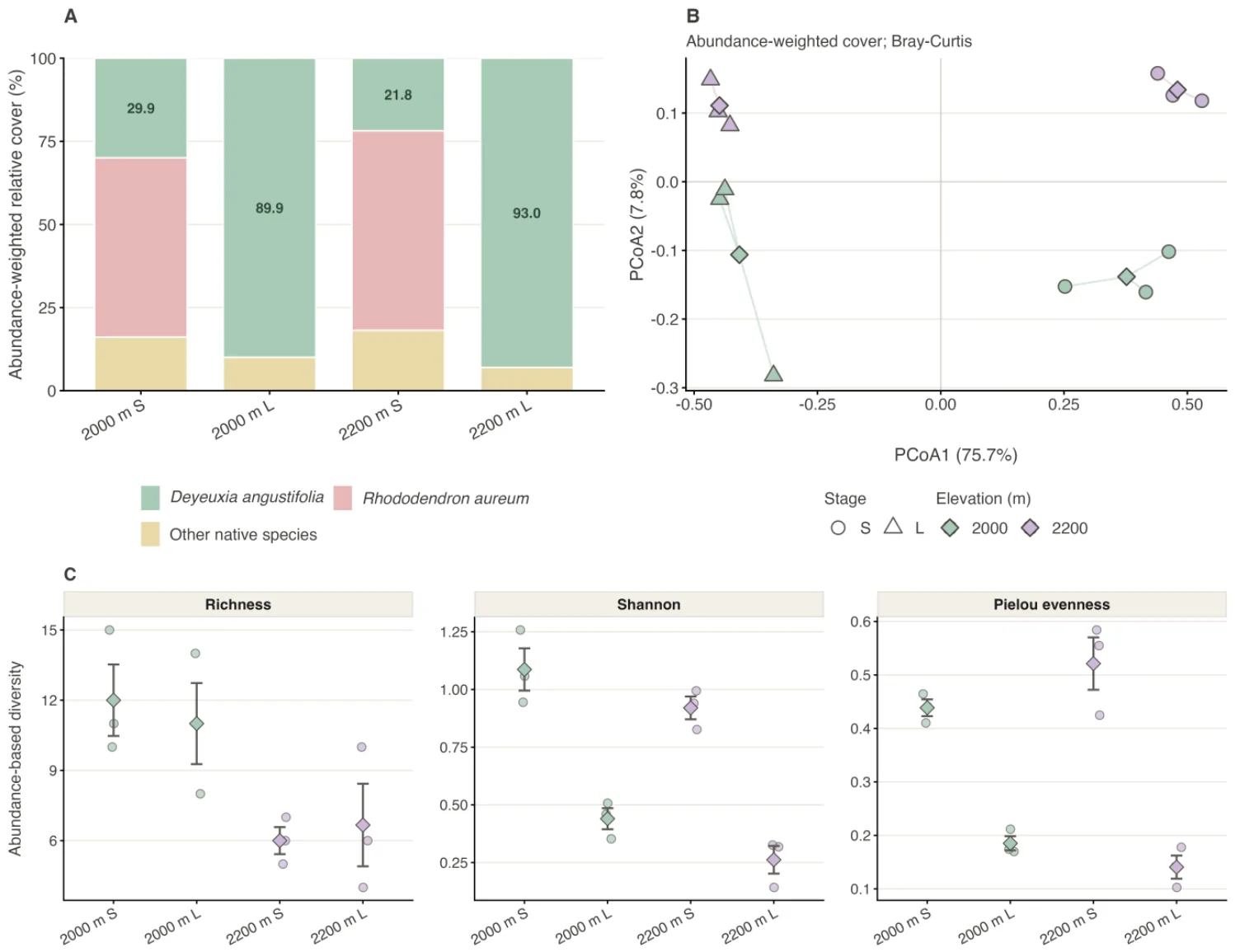
Plant community structure across elevations and encroachment stages. (**A**) Mean abundance-weighted relative cover of *D. angustifolia*, *R. aureum*, and other native species in four habitats. For each species in each plot, weighted cover was calculated as abundance × single-plant cover, and relative cover was its percentage of the summed weighted cover of all species in that plot. Bars show habitat means based on three replicate plots (*n* = 3); numbers within the green segments denote the mean relative cover (%) of *D. angustifolia*. (**B**) Principal coordinates analysis (PCoA) of Bray–Curtis dissimilarities calculated from the abundance-weighted species cover matrix. (**C**) Abundance-based species richness, Shannon diversity, and Pielou evenness. Points represent individual plots, diamonds represent habitat means, and error bars are ±SE (*n* = 3 plots per habitat). S and L denote slight and large encroachment, respectively.

Following abundance-weighted recalculation of relative cover, plant community structure differed markedly among elevations and encroachment stages (Figure 1). The mean relative cover of *D*. *angustifolia* increased from 29.94 ± 5.79% (mean ± SE) in the 2000 m slight-encroachment habitat (S) to 89.94 ± 0.38% in the corresponding large-encroachment habitat (L). At 2200 m, its relative cover increased from 21.84 ± 1.39% in S to 93.01 ± 2.41% in L (*n* = 3 plots per habitat; Figure 1A). The PCoA based on Bray–Curtis dissimilarities of the abundance-weighted species cover matrix showed clear separation among the four habitats, with the first two axes explaining 75.7% and 7.8% of the compositional variation, respectively (Figure 1B). Abundance-based Shannon diversity and Pielou evenness were lower in L than in S habitats at both elevations (Figure 1C). At 2000 m, Shannon diversity declined from 1.087 ± 0.092 in S to 0.440 ± 0.046 in L, while Pielou evenness declined from 0.439 ± 0.016 to 0.185 ± 0.013. At 2200 m, Shannon diversity declined from 0.920 ± 0.049 to 0.262 ± 0.060, and Pielou evenness declined from 0.521 ± 0.049 to 0.141 ± 0.022. Collectively, these results show that increasing local dominance of *D. angustifolia* was associated primarily with compositional replacement and marked reductions in community diversity and evenness, rather than a consistent decline in species richness.

### 2.2. Growth Trait Trajectories and Morphological Differentiation Among the Selected D. angustifolia Habitats

**Figure 2:**
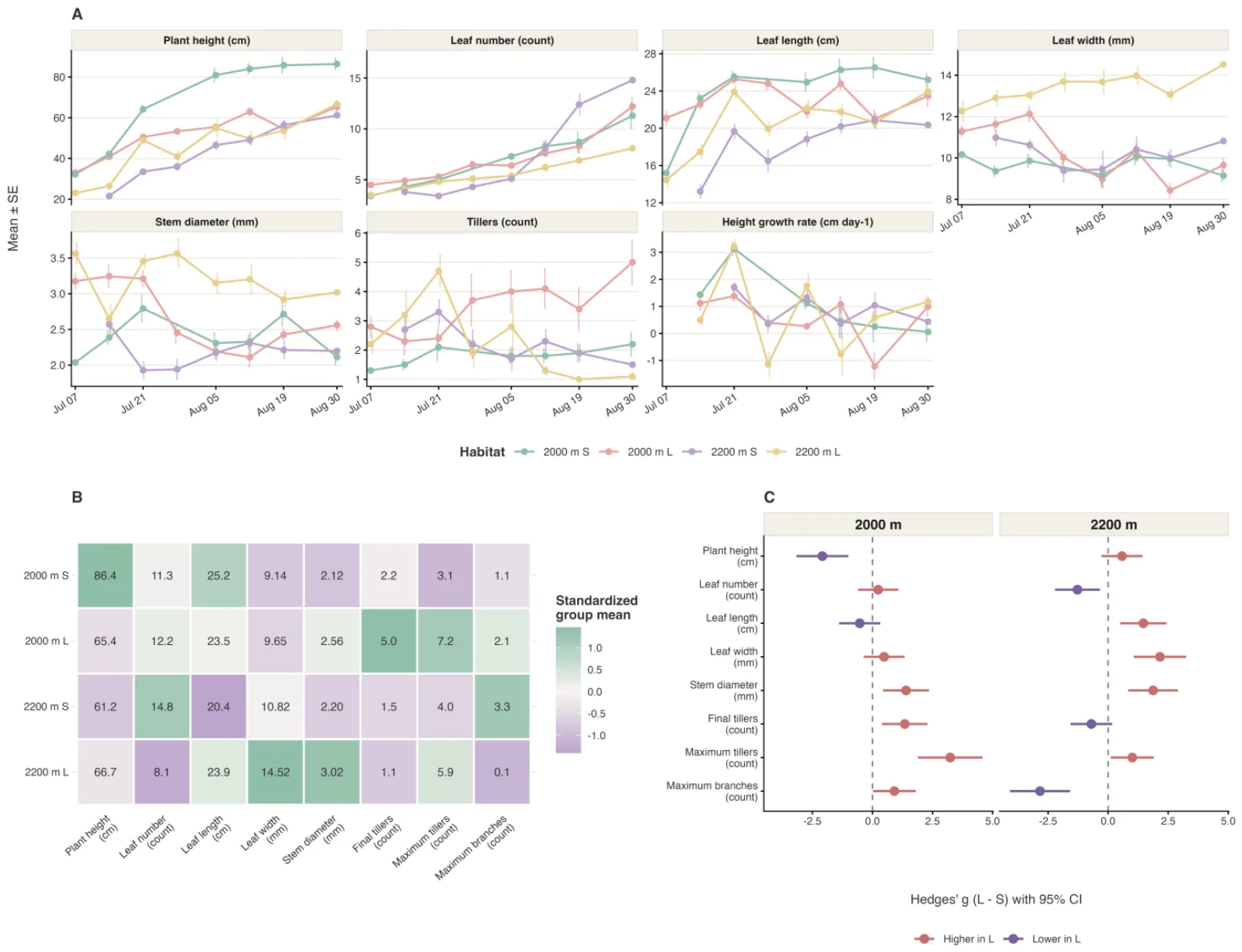
Seasonal growth trajectories and morphological trait differentiation of *D. angustifolia* across the four selected alpine tundra habitats. (**A**) Temporal trajectories of plant height, leaf number, leaf length, leaf width, stem diameter, tiller number, and individual height growth rate. Points and error bars show mean ± SE of the available marked individuals (normally *n* = 10 per habitat–date combination). Height growth rate was calculated between consecutive available observations for each individual. (**B**) Trait-wise standardized habitat means. Plant height, leaf number, leaf length, leaf width, stem diameter, and final tiller number were measured on 30 August; maximum tiller and branch numbers were derived across the available census dates. Numbers in cells are unstandardized means, whereas colors represent trait-wise z-scores across the four habitat means. (**C**) Hedges’ *g* and 95% confidence intervals for the L–S contrast within each elevation. Positive values indicate higher means in L and negative values indicate lower means in L. Each contrast is based on ten individuals per habitat.

The detailed phenological records comprised ten marked individuals in each of four selected elevation × encroachment habitats (40 individuals in total), repeatedly monitored across six census dates from 7 July to 30 August during the 2023 growing season (Figure 2A). Repeated-measures models incorporating individual-level random intercepts revealed strong temporal and spatial modulation of vegetative growth (Supplement Table S2A). Significant elevation × encroachment stage × date three-way interactions were detected for continuous architectural traits, including plant height (*p* = 0.001719), leaf width (*p* = 1.01 × 10^−5^), and stem diameter (*p* = 1.94 × 10^−7^). Individual height growth rate peaked in mid-to-late July across all habitats (1.5–3.2 cm day^−1^) before decelerating toward late August.

At the end of season vegetative plateau (30 August 2023), two-way factorial ANOVA on endpoint traits (*n* = 40) confirmed substantial structural divergence driven by elevation, encroachment stage, and their interaction (Supplement Table S2B). Significant elevation × stage interactions were detected for plant height (*p* < 0.001), leaf number (*p* = 0.009), leaf length (*p* = 0.004), leaf width (*p* < 0.001), active tillers (*p* = 0.001), seasonal maximum tillers (*p* = 0.030), and seasonal maximum branch number (*p* < 0.001).

Standardized group means (Z-scores; Figure 2B) and effect size contrasts (Hedges’ g with 95% confidence intervals for L–S; Figure 2C; Supplement Table S2C) delineated distinct elevation-dependent morphological adjustments. At lower elevation (2000 m): L adopted a compact, highly tilled clonal architecture. Compared with the corresponding S habitat, 2000 m L individuals had significantly shorter vegetative height (65.39 ± 3.27 vs. 86.40 ± 2.82 cm, mean ± SE; Hedges’ g = −2.08, 95% CI: −3.17 to −1.00), thicker stems (2.56 ± 0.08 vs. 2.12 ± 0.11 mm; g = 1.40, 95% CI: 0.44 to 2.35), more active final tillers (5.00 ± 0.79 vs. 2.20 ± 0.42; g = 1.34, 95% CI: 0.40 to 2.29), and higher seasonal maximum tillers (7.20 ± 0.44 vs. 3.10 ± 0.31; g = 3.24, 95% CI: 1.89 to 4.59). Branch number also trended higher in L (2.10 ± 0.28 vs. 1.10 ± 0.38; g = 0.91, 95% CI: 0.02 to 1.80), whereas differences in leaf length (g = −0.53), leaf width (g = 0.49), and leaf number (g = 0.24) were not statistically resolved. At the higher elevation (2200 m): L shifted biomass allocation toward foliar expansion and individual culm reinforcement rather than lateral tillering. Individuals in 2200 m L exhibited markedly greater final leaf width (14.52 ± 0.33 vs. 10.82 ± 0.65 mm; g = 2.16, 95% CI: 1.06 to 3.25), thicker stems (3.02 ± 0.14 vs. 2.20 ± 0.12 mm; g = 1.87, 95% CI: 0.83 to 2.91), longer leaves (23.94 ± 0.45 vs. 20.36 ± 0.94 cm; g = 1.47, 95% CI: 0.50 to 2.43), and higher seasonal maximum tillers (5.90 ± 0.72 vs. 4.00 ± 0.37; g = 1.01, 95% CI: 0.10 to 1.91). Conversely, 2200 m L individuals produced significantly fewer leaves (8.10 ± 0.35 vs. 14.80 ± 2.22; g = −1.28, 95% CI: −2.21 to −0.34) and nearly suppressed branching (0.10 ± 0.10 vs. 3.30 ± 0.47; g = −2.84, 95% CI: −4.09 to −1.59). Confidence intervals for final plant height (g = 0.58, 95% CI: −0.28 to 1.44) and final active tillers (g = −0.70, 95% CI: −1.57 to 0.17) crossed zero at 2200 m.

These coordinated morphological contrasts demonstrate contrasting phenotypic profiles between the two elevation zones: lateral tiller proliferation and compact shoot clustering at 2000 m versus individual structural culm reinforcement at 2200 m.

### 2.3. Soil Nutrient Status and Enzyme Activities Differed Between Slightly and Largely Encroached Habitats

**Figure 3:**
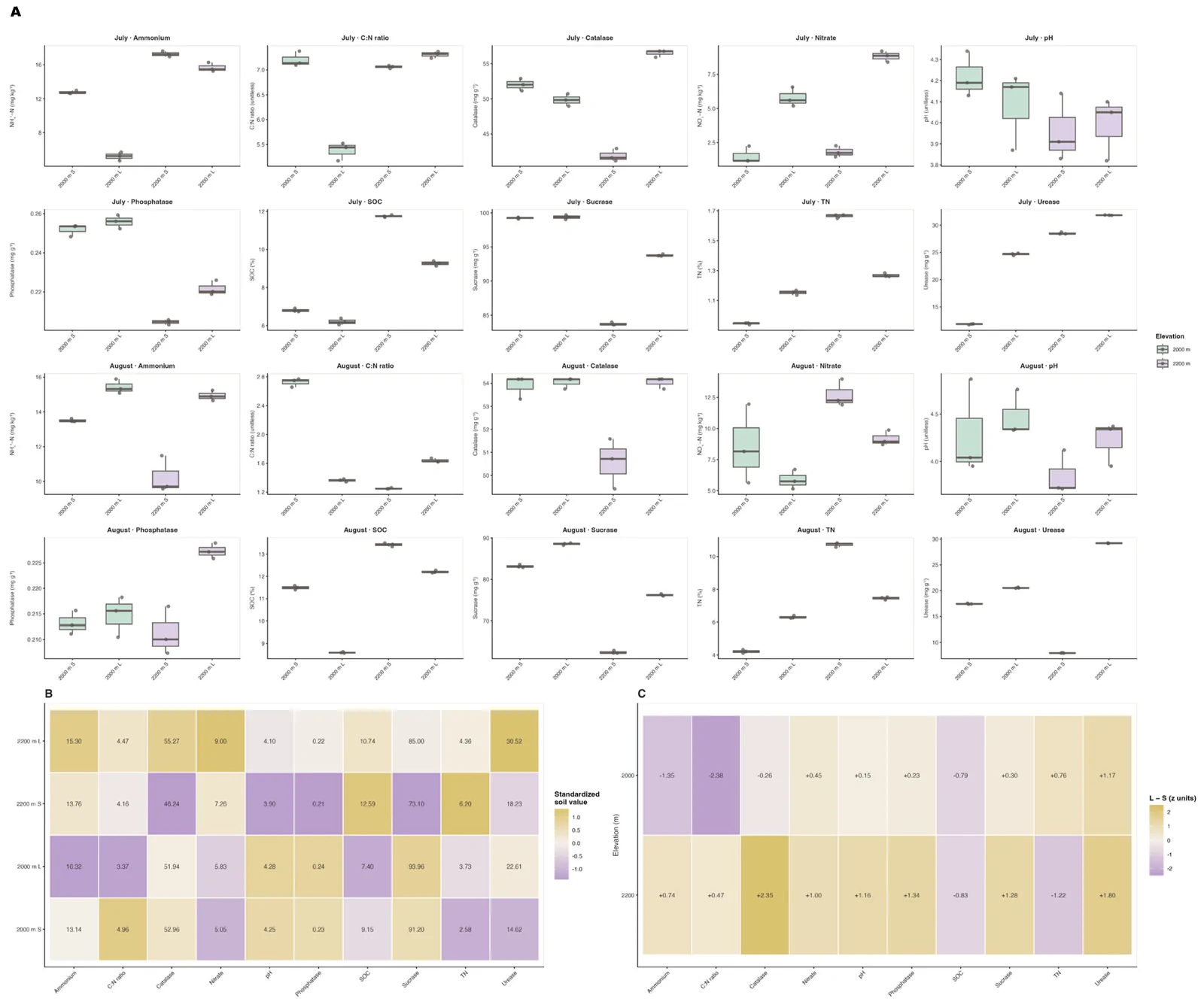
Phenology-matched soil carbon–nitrogen environment and enzyme activities. (**A**) soil C-N-related properties, inorganic nitrogen pools and soil enzyme activities. (**B**) Standardized heatmap of soil variables. (**C**) Standardized large-minus-slight contrast (L–S) within each elevation.

Phenology-matched soil data showed clear differences in soil physicochemical properties and enzyme activities among elevation and expansion-stage habitats (Figure 3). At 2000 m, largely expanded plots (L) had lower SOC than slightly expanded plots (S), but relatively higher TN, nitrate nitrogen and urease activity. At 2200 m, largely expanded plots (L) also showed higher nitrate nitrogen, ammonium nitrogen, urease activity and sucrase activity, whereas SOC and TN were relatively lower. Three-way ANOVA indicated that expansion stage had significant effects on SOC, TN, C:N ratio, nitrate nitrogen, ammonium nitrogen and multiple enzyme activities. These results suggest that soil nutrient status and enzyme-mediated transformation processes differed substantially between expansion habitats.

To verify whether belowground soil properties resulted from *D. angustifolia* presence and litter accumulation rather than pre-existing micro-site edaphic anomalies, soil properties across encroachment gradients (S and L) were benchmarked against the unencroached native tundra baseline (R/CK, 0% *D. angustifolia* cover; Figure S1). Soil organic carbon (SOC) and total nitrogen (TN) pools exhibited pronounced elevation- and season-dependent trajectories relative to the native baseline. At 2000 m, baseline R (CK) soils maintained relatively low and stable pools in July. Encroachment led to dynamic mid-growing season increases, with SOC peaking at the S stage in August before moderating at the L stage, while TN steadily increased from R/CK to L in August. At 2200 m, pristine native tundra (R/CK) stored substantial baseline carbon and nitrogen pools during early summer (July). Across the encroachment gradient, July SOC and TN experienced continuous depletion toward heavily encroached patches, demonstrating that *D. angustifolia* did not preferentially establish in pre-existing high-fertility hotspots.

### 2.4. Soil Bacterial Diversity and Community Composition Varied with Elevation, Expansion Stage and Month

**Figure 4:**
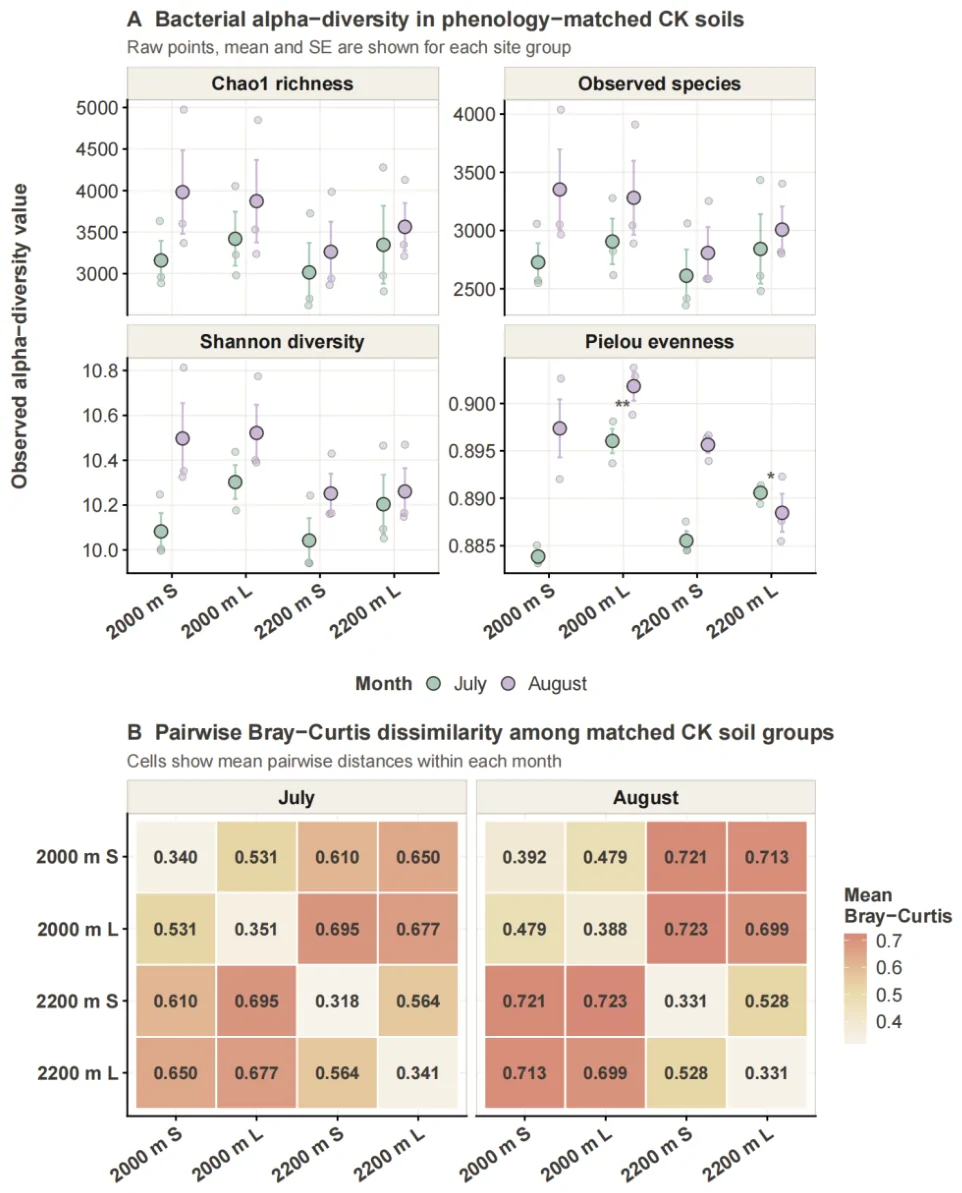
Soil bacterial alpha diversity and community dissimilarity across elevation, encroachment stage, and month. (**A**) Bacterial alpha-diversity indices (Chao1 richness, observed species, Shannon diversity, and Pielou evenness) across the four spatial habitat states (2000 m S, 2000 m L, 2200 m S, and 2200 m L) in July (green circles) and August (purple circles) in phenology-matched unamended (CK) soils. Pale circles represent raw sample observations, larger filled circles indicate group means, and error bars denote standard errors (±SE). Pairwise comparisons evaluate slight (S) versus large (L) encroachment states within each elevation × month cell using Welch’s *t*-tests (Table S3A). * and ** denote unadjusted Welch-test *p* values of <0.05 and <0.01, respectively. (**B**) Pairwise Bray–Curtis dissimilarity heatmaps showing mean bacterial community compositional distances among the four habitat groups evaluated separately in July (left panel) and August (right panel). Values within cells represent mean pairwise Bray–Curtis distances, with darker shades indicating greater compositional divergence. S and L denote slightly and largely encroached habitats, respectively. Full global PERMANOVA and multivariate dispersion (PERMDISP) statistics are reported in Table S3B,C.

Bacterial alpha diversity did not show consistently strong differences between slightly and largely expanded plots (Figure 4A). Chao1 richness, observed species and Shannon diversity showed relatively weak differences between expansion stages, whereas Pielou evenness differed more clearly in July. Compared with alpha diversity, bacterial community composition was more sensitive to habitat differences. PERMANOVA based on Bray–Curtis dissimilarity showed that elevation was the dominant factor explaining bacterial community variation (R^2^ = 33.3%, *p* = 0.001), while expansion stage (R^2^ = 9.0%, *p* = 0.001) and month (R^2^ = 9.5%, *p* = 0.001) also contributed significantly. The interactions of elevation × expansion stage, elevation × month and expansion stage × month were significant, indicating that bacterial community composition was jointly associated with spatial habitat differences and seasonal variation.

### 2.5. Decomposition of D. angustifolia Litter

**Figure 5:**
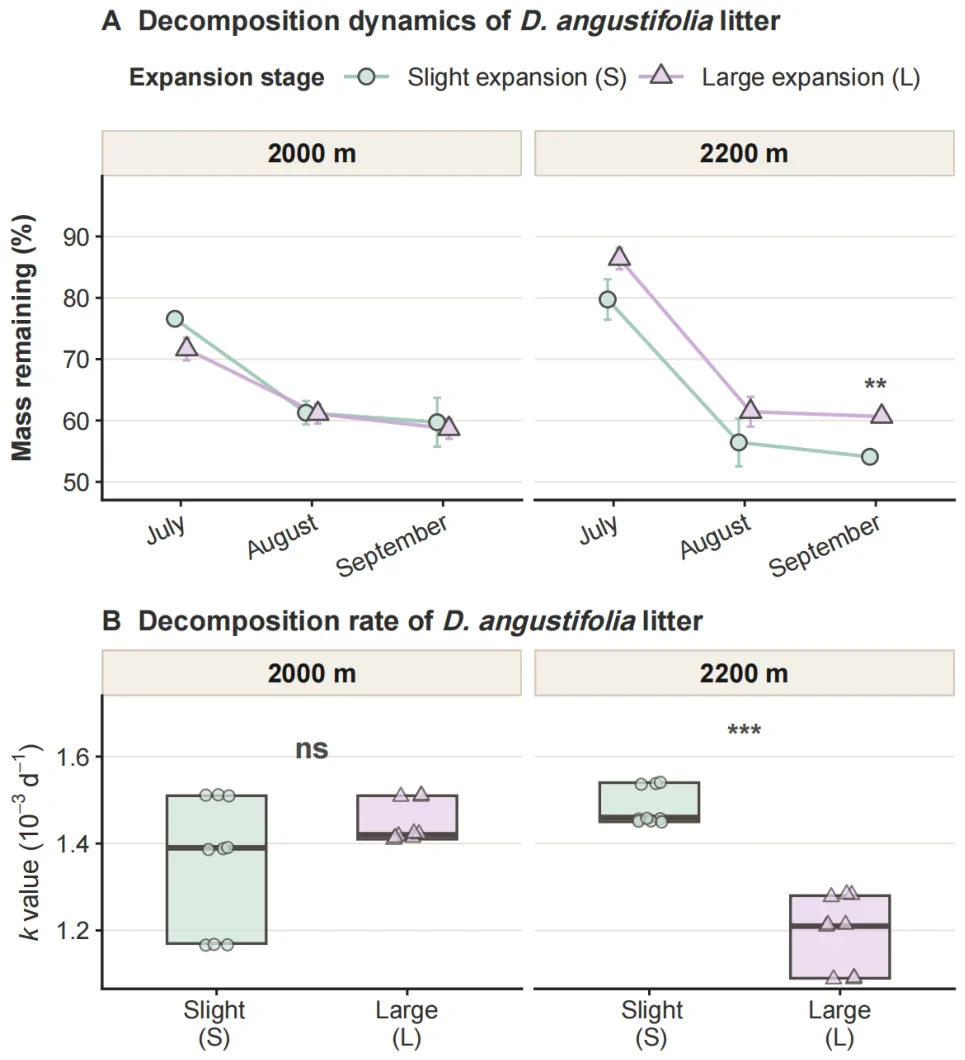
Decomposition dynamics of *D. angustifolia* litter in slightly and largely encroached habitats. (**A**) Temporal dynamics of litter mass remaining at 2000 m and 2200 m across July, August and September, shown as mean ± SE. (**B**) Decomposition rate constant (k) of *D. angustifolia* litter between S and L habitats, shown as 10^−3^ d^−1^. Significance marks are based on Welch’s *t*-tests. ** *p* < 0.01, *** *p* < 0.001; ns, not significant.

The mass remaining of *D. angustifolia* litter decreased with decomposition time, but differences between slightly and largely encroached habitats (S and L) were elevation-dependent (Figure 5). At 2000 m, litter mass remaining declined from 76.58% in July to 59.72% in September in slightly expanded plots (S), and from 71.63% to 58.65% in largely expanded plots. No significant difference was detected between expansion stages within any sampling month at this elevation (*p* > 0.05). At the higher encroachment front of 2200 m, mass remaining declined from 79.73% to 54.08% in slightly expanded plots, whereas it declined from 86.38% to 60.65% in largely expanded plots (L). In September, mass remaining was significantly higher in largely expanded plots than in slightly expanded plots (*p* = 0.002).

The decomposition rate constant further supported this pattern. At 2000 m, k values of *D. angustifolia* litter were 1.357 × 10^−3^ d^−1^ in slightly expanded plots (S) and 1.447 × 10^−3^ d^−1^ in largely expanded plots (L), with no significant difference between expansion stages (*p* = 0.117). In contrast, at 2200 m, k value was significantly higher in slightly expanded plots than in largely expanded plots (1.483 × 10^−3^ vs. 1.193 × 10^−3^ d^−1^; *p* < 0.001). The linear model showed a significant effect of expansion stage on k value (*p* = 0.003) and a significant elevation × expansion-stage interaction (*p* < 0.001). These results indicate that decomposition of *D. angustifolia* litter was more strongly constrained in largely encroached habitats at the high-elevation encroachment front.

## 3. Discussion

**Figure 6:**
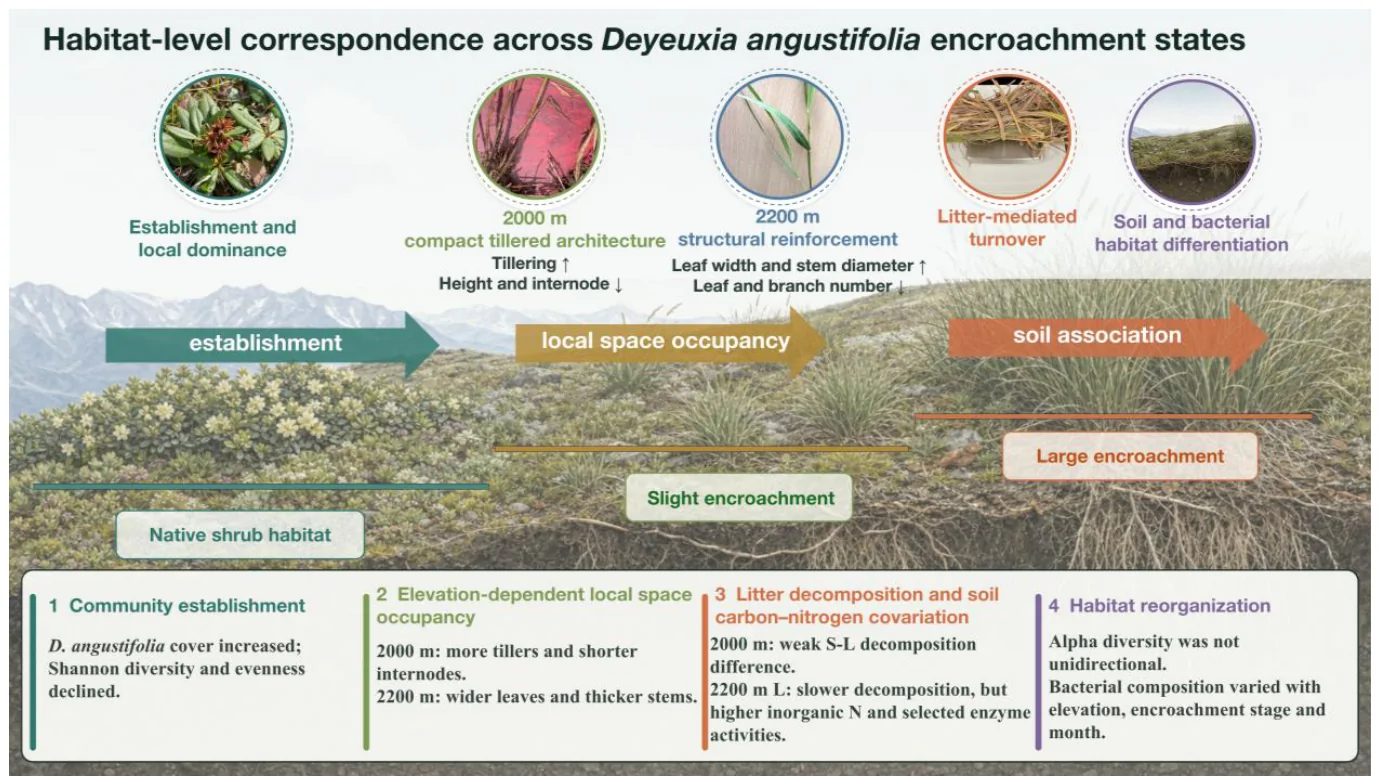
Conceptual synthesis of habitat reorganization during *D. angustifolia* encroachment in Changbaishan alpine tundra (all field photographs displayed in Figure 6 were taken on-site by the authors).

The encroachment of *D. angustifolia* into the Changbaishan alpine tundra is not merely an increase in graminoid cover [53,54]. By integrating plant community surveys, growth trait monitoring, litter decomposition, soil physicochemical properties, enzyme activities, and bacterial community data, this study demonstrates that slightly and largely encroached habitats differ systematically in aboveground vegetation structure, litter mass loss, inorganic nitrogen availability, enzyme activities, and bacterial community composition (Figure 6).

According to microbial nitrogen mining theory, high availability of readily accessible inorganic nitrogen (NH_4_-N and NO_3_-N) suppresses microbial synthesis of costly oxidative enzymes needed to break down recalcitrant lignocellulosic compounds. At 2200 m, heavily encroached soils maintained high inorganic nitrogen levels throughout the summer (NH_4_-N = 14.93 mg/kg; NO_3_-N = 9.18 mg/kg). Under this elevated mineral nitrogen regime, microbes do not need to mine recalcitrant organic matter for nitrogen, which suppresses late-stage decomposition of *D. angustifolia* foliar residues (60.65% mass remaining in September at 2200 m L vs. 54.08% at 2200 m S; k = 1.19 × 10^−3^ per day vs. 1.48 × 10^−3^ per day).

Substrate quality exerts a dominant control on initial and intermediate decomposition stages. At 2200 m, *D. angustifolia* invested heavily in structural leaf and stem reinforcement, displaying substantially wider leaves (14.52 mm) and thicker stems (3.02 mm) than at 2000 m (9.65 mm and 2.30 mm, respectively). Foliage produced under harsher high-elevation conditions typically contains higher concentrations of structural cutins, condensed tannins, and lignin, as well as a higher C:N ratio. This intrinsic biochemical recalcitrance provides a direct substrate-level barrier to microbial catabolism.

The 2200 m encroachment front is subjected to intense environmental filtering, characterized by lower ambient temperatures, persistent wind exposure, and recurrent freeze–thaw cycles. Cold temperature constraints directly reduce extracellular enzyme kinetics and suppress microbial catabolic activity. Consequently, slower mass loss at 2200 m is partly an abiotic thermodynamic consequence of high-elevation alpine climates.

The observed surge in inorganic nitrogen at 2200 m L likely stems from a mismatch between gross nitrogen mineralization and microbial/plant uptake: low thermal energy suppresses active assimilation and nitrification, allowing for mineralized ammonium to accumulate in the shallow topsoil. Elevated alkaline phosphatase (0.227 mg phenol/g) and urease activities (29.20 mg NH_4_-N/g) at 2200 m L indicate heightened functional demand for organic phosphorus and nitrogen mineralization in nutrient-poor volcanic soils. Simultaneously, elevated catalase activity (54.04–56.49 mL/g) reflects an adaptive microbial antioxidant response to reactive oxygen species (ROS) induced by cold and wind exposure.

Rather than an isolated vegetation shift, *D. angustifolia* encroachment is associated with a coupled pattern of aboveground morphological trait adjustment, local dominance, and belowground differentiation linked to litter decomposition dynamics and associated soil habitat.

Differences in community structure delineate these spatial habitat contrasts. Largely encroached plots showed greater relative cover of *D. angustifolia* alongside reduced dominance of *R. aureum* and other native species, indicating that *D. angustifolia* has formed locally dominant patches within the native tundra matrix. Given the strong environmental filtering imposed by low temperatures, short growing seasons, and nutrient-poor soils in alpine tundra, the establishment of such dominant patches represents a notable ecological boundary shift. In this observational design, slightly encroached habitats (S) and largely encroached habitats (L) represent distinct spatial states along an encroachment gradient, providing a comparative framework to evaluate associated plant traits and soil properties.

Across this spatial gradient, the growth traits of *D. angustifolia* exhibited clear elevation-dependent variation. At 2000 m, largely encroached habitats were characterized by increased tiller numbers and shorter internodes. Under the comparatively milder microclimatic conditions at lower elevation, producing more tillers and shorter internode spacers results in a tighter, clustered growth form that facilitates spatial occupancy within local patches. At 2200 m, by contrast, largely encroached habitats showed wider leaves and thicker stems, alongside reduced branch and leaf counts. This morphological response reflects greater biomass allocation to individual stem support and leaf area rather than tiller multiplication. Thus, *D. angustifolia* does not display a uniform morphological response across its range; instead, it exhibits distinct, elevation-specific trait adjustments, characterized by increased tillering and compact shoot architecture at lower elevation, and leaf widening with stem thickening at the higher-elevation front.

This trait transition matters ecologically because it marks the point where *D. angustifolia* moves from entering a community to reshaping its local resource environment. Denser tillering and shorter internodes build a more continuous canopy and a tighter clonal network, improving the capture of light, space and soil resources within a patch; wider leaves and thicker stems, in turn, support individual performance where low temperature and a short growing season constrain growth. Read together, these trait patterns indicate that the encroachment front advances not passively but through growth strategies tuned to elevation-specific environmental pressure.

As *D. angustifolia* shifts from slight establishment to local dominance, litter becomes the medium that links aboveground expansion to belowground processes. Annual litter production was not directly quantified here, so largely encroached habitats cannot be described as having a demonstrated increase in litter input, but higher cover with the decomposition results indicate that *D. angustifolia* litter becomes more relevant to local soil processes once dominance is established. Decomposition itself was not uniform across elevations: at 2000 m it differed only weakly between slightly and largely encroached habitats, whereas at 2200 m largely encroached habitats showed slower decomposition and greater mass remaining. Local dominance at the high-elevation front therefore does not automatically translate into faster nutrient release.

This elevation dependence of litter decomposition offers a more coherent explanation for the soil nutrient patterns than dominance alone would. At 2000 m, milder conditions may let *D. angustifolia* litter enter organic matter turnover relatively quickly, so clonal expansion and shifts in nitrogen transformation indicators tend to co-occur. At 2200 m, slower decomposition coincided with higher inorganic nitrogen availability and elevated activity of several enzymes—a combination suggesting a more complex belowground state in which litter residues accumulate even as local nitrogen transformation and enzymatic processing remain active. This “slow decomposition, locally active nutrient transformation” signature may be precisely what distinguishes the high-elevation encroachment front from lower-elevation patches. The fine mesh size of litterbag (0.2 mm) physically excludes macrofauna (e.g., earthworms) and most mesofauna (e.g., larger mites and collembolans). Consequently, the recorded litter mass loss reflects predominantly microbial decomposition and leaching, rather than whole-soil food web fragmentation.

Bacterial community composition adds a further layer to this picture of belowground differentiation. Alpha-diversity indices showed no consistent, unidirectional response between slightly and largely encroached habitats, so diversity alone cannot summarize the microbial response. Bray–Curtis dissimilarities, however, revealed clear compositional separation among elevation and encroachment-stage groups, pointing to restructuring of bacterial assemblages rather than a simple gain or loss of diversity. Because bacterial communities participate directly in litter decomposition, nitrogen transformation and organic matter turnover, this compositional shift is plausibly an integral part of the soil habitat differentiation that accompanies *D. angustifolia* expansion.

**Figure 7:**
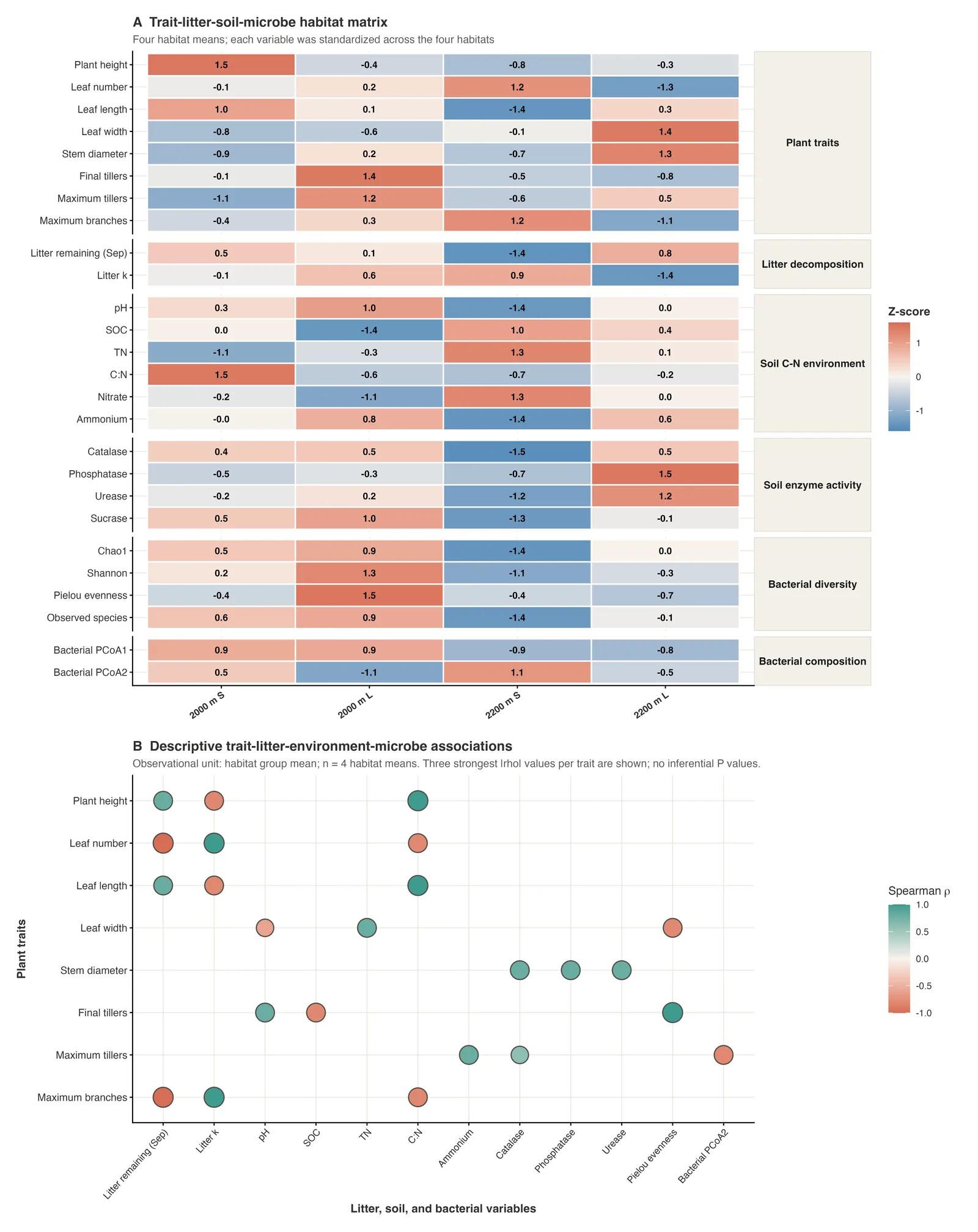
Integrated habitat-level matrix and descriptive rank associations among plant growth traits, litter decomposition, soil properties, and bacterial communities. (**A**) Standardized habitat matrix across the four established spatial habitat groups (2000 m S, 2000 m L, 2200 m, and 2200 m L). Cell numbers and color shading indicate variable-wise standardized *Z*-scores computed across the four habitat group means. (**B**) Descriptive Spearman rank correlation matrix (ρ) evaluating pairwise associations between plant morphological traits and litter, soil, and bacterial variables. Note on statistical interpretation: The observational unit in panel (**B**) is the habitat-level group mean. (Circle size and color gradients represent the magnitude and direction of Spearman rank correlation coefficients (ρ). This panel is presented strictly as an exploratory, descriptive synthesis of habitat-level covariation across spatial encroachment states, rather than sample-level inferential hypothesis testing or direct causal proof).

Taken together, the study integrated matrix of plant growth traits, litter mass loss, soil physicochemical properties, and bacterial community composition delineates a coordinated aboveground–belowground habitat differentiation along the *D. angustifolia* encroachment gradient (Figure 7). Rather than an isolated shift in vegetation cover, *D. angustifolia* encroachment across elevations is characterized by distinct morphological trait profiles corresponding to localized shifts in soil nutrient pools, enzyme activities, and bacterial community turnover. At lower elevation (2000 m), the spatial contrast between slight and large encroachment states corresponds to increased tiller production alongside higher soil pH, active nitrogen availability, and bacterial diversity indices. Conversely, at the high-elevation front (2200 m), large encroachment is characterized by leaf widening and stem thickening, accompanied by slower litter mass turnover, higher litter residue accumulation in late autumn, and elevated phosphatase and urease activities. The observed multivariate correspondence elevates *D. angustifolia* encroachment from a simple descriptive change in species cover to an integrated ecological process linking plant morphological adjustment with belowground soil and microbial habitat differentiation.

These findings provide habitat-level insights into the ongoing meadowization of the Changbaishan alpine tundra. When viewed through vegetation surveys alone, meadowization appears as the gradual replacement of dwarf shrubs by graminoids; however, our findings demonstrate that this vegetative shift is accompanied by divergent litter decomposition dynamics, restructured soil carbon–nitrogen pools, and distinct bacterial community assemblages. These coordinated above and below ground shifts suggest that soil habitat divergence occurs in tandem with *D. angustifolia* patch dominance rather than an entirely decoupled downstream consequence.

Nonetheless, because our cross-sectional study was conducted within a single growing season and habitat-level associations are based on four composite ecological states, these relationships represent descriptive covariation across spatial gradients rather than demonstrated temporal causality. Future studies integrating multi-year demographic tracking, root exudate characterization, total belowground biomass allocation, and fungal metagenomics across encroachment boundaries will be essential to rigorously test the mechanistic pathways underlying these plant–soil–microbe linkages. We acknowledge that comparing spatial encroachment gradients within a single growing season provides habitat-level associations rather than direct evidence of temporal succession. Pre-existing micro-environmental variation could also facilitate initial *D. angustifolia* establishment. Long-term permanent plot tracking, coupled with reciprocal soil transplantation and isotopic tracing, will be essential to definitively establish temporal causality and untangle pre-existing edaphic biases from plant-induced soil conditioning.

## 4. Materials and Methods

### 4.1. Study Site

This study was conducted in the alpine tundra of Changbaishan, Jilin Province, northeastern China [14,28]. Changbaishan alpine tundra is a middle to low-latitude tundra ecosystem characterized by low temperature, a short growing season, shallow soils and strong environmental filtering [52,55]. The mean annual temperature (MAT) is −7.3 °C, with the coldest month (January) averaging below −20 °C and the warmest month (July) averaging 8.0–10.0 °C. Mean temperatures during the plant growing season (June to September) range between 3.37 °C and 8.82 °C. Mean annual precipitation (MAP) spans 558.7–1553.7 mm, with precipitation heavily concentrated from July through September. The region is characterized by severe wind exposure, having a mean annual wind speed of 11.7 m/s, over 280 gale days per year (≥Beaufort force 8), and extreme wind speeds up to 40 m/s recorded across 10 months annually. Total annual solar radiation ranges from 5.07 to 5.12 MJm^−2^ [10,27,56].

The soil is classified as Mountain Tundra Soil, developed on volcanic ejecta and basaltic pyroclastic materials. The soil profile is skeletal and shallow (10–20 cm total thickness), comprising a 0–5 cm organic transition/humus horizon, a 5–10 cm gravelly humus layer, and an underlying volcanic bedrock layer (10–30 cm). Two target elevation belts were investigated: the lower alpine tundra belt at 2000 m a.s.l. (2022 m actual, 41°59′15.06″, 128°00′18.28″) and the higher-elevation encroachment front at 2200 m a.s.l. (2223 m actual, 41°59′29.40″, 128°01′03.42″) [21,22].

In recent decades, the upward expansion of *D. angustifolia* has become increasingly evident in this region, forming dense graminoid patches along the alpine tundra encroachment front and altering native shrub-dominated communities [18,19,27,57]. Taxonomic classification and botanical nomenclature in this study follow the regional treatments in the *Flora of China (Vol. 22)* and *Flora Reipublicae Popularis Sinicae (Vol. 9(3))*. This species was originally described as *Calamagrostis angustifolia* Kom. and subsequently transferred to *Deyeuxia angustifolia* (Kom.) Y.L.Chang; both names are treated synonymously, and *Deyeuxia angustifolia* (Kom.) Y.L.Chang is adopted here in accordance with East Asian alpine flora.

Two elevation belts were selected: 2000 m, representing the lower alpine tundra zone, and 2200 m, representing the higher-elevation encroachment front. At each elevation, slightly and largely encroachment (S and L) *D. angustifolia* habitats were identified according to field cover and dominance of *D. angustifolia*. Four habitat types were therefore included: 2000 m slight expansion, 2000 m large expansion, 2200 m slight expansion and 2200 m large expansion.

Slight encroachment (S): Defined as habitats where *D. angustifolia* occurred as scattered patches with a relative cover of 10–40% within the native dwarf shrub matrix.

Large encroachment (L): Defined as contiguous, heavily encroached patches where *D. angustifolia* achieved absolute dominance with a relative cover of ≥70% [18,22,52].

At each elevation, three independent 20 m × 20 m replicate plots with similar topographic attributes (slope 8–9°, southwest aspect, and minimal anthropogenic disturbance) were selected for each encroachment stage (2 elevations × 2 encroachment stages × 3 independent replicate plots = 12 plots in total).

### 4.2. Experimental Design and Plant Community Survey

A habitat-level nested experimental design was implemented across the 12 established plots. Plant community surveys were conducted within the unamended control plots (CK, without litter addition) during the 2023 growing season to avoid disturbance from artificial litter manipulations. In each habitat type, three replicate 10 m × 10 m quadrats were surveyed (*n* = 12 quadrats in total).

Within each 10 m × 10 m quadrat, all vascular plant species were identified, and their abundance, total cover, height (maximum, mean, and minimum), and crown width (maximum, mean, and minimum) were systematically recorded. For the target species *D. angustifolia*, specific parameters including ramet count, relative cover, vegetative height, crown diameter, and root length were measured. Community *alpha*-diversity was quantified using species richness, Shannon diversity, and Pielou evenness, while community compositional dissimilarity across habitats was evaluated via Bray–Curtis distance matrices.

### 4.3. Phenological Monitoring and Growth Trait Measurements

Continuous phenological and morphological monitoring of *D. angustifolia* was carried out from 7 July to 30 August 2023, with an observation frequency of every 7–8 days. Within each of the 12 plots, 10 representative, healthy, mature individuals of *D. angustifolia* exhibiting uniform vigor were randomly selected for non-destructive tracking (*n* = 10 individuals per plot × 12 plots = 120 individuals per census). Growth traits included plant height, leaf number, leaf length, leaf width, stem diameter, tiller number, maximum tiller number, and branch number.

These traits were selected to represent different aspects of plant performance. Plant height and leaf traits reflect aboveground resource acquisition, whereas tiller number, and branch number are related to clonal propagation and spatial expansion. For integrated analyses with soil and bacterial data, trait observations from months corresponding to the soil sampling period were used.

### 4.4. Litter Decomposition Analysis

In September 2023, during the plant senescent period in the alpine tundra, current-year aboveground foliar litter of *D. angustifolia* was collected in situ across designated plots. Litter samples were oven-dried at 65 °C to constant mass to determine initial dry weight. One subset of dried litter was stored in glass desiccators with silica gel for baseline chemical characterization, while the remainder was used for in situ decomposition.

Dried foliar litter (20.00 g per bag) was sealed into nylon mesh litterbags (15 cm × 15 cm, mesh size 0.2 mm). In each of the 12 field plots (2 elevations × 2 encroachment stages × 3 replicate plots), 15 litterbags were placed flat in direct contact with the mineral topsoil and fixed with bamboo pegs (180 bags in total). Litterbags were retrieved on 2 July, 16 August, and 29 September 2024. At each sampling date, 3 litterbags were randomly retrieved from each plot and combined into a single homogeneous composite sample per plot (2 elevations × 2 encroachment stages × 3 sampling dates × 3 plot replicates = 36 composite litter samples in total). This pooling protocol was implemented to maintain direct methodological consistency with the underlying soil cores, which were similarly composited beneath the 3 retrieved litterbags to obtain sufficient soil mass from the shallow, skeletal volcanic soil layer (0–10 cm) for physicochemical, enzymatic, and molecular assays [58].

After retrieval, litter samples were dried to constant mass and weighed. The mass remaining ratio was calculated as the ratio of remaining dry mass to initial dry mass. The decomposition rate constant was calculated using a single exponential decay model [34,59,60,61]:

$$k=-\frac{\ln(M/M_{0})}{t}$$

where *M*_0_ is the initial litter mass, *M* is the remaining litter mass at time *t*, and *t* is decomposition time in days. In the present manuscript, only *D. angustifolia* litter in slightly and largely encroachment habitats (S and L) was used to evaluate expansion-stage differences in decomposition.

### 4.5. Soil Sampling, Physicochemical Properties, and Enzymatic Assays

Soil sampling was conducted simultaneously with litterbag retrievals across the three growing season months (July, August, and September 2024). Two distinct sets of soil samples were collected:

Soil beneath litterbags: In litter-amended plots, soil cores (0–10 cm depth) directly beneath each retrieved litterbag were collected (3 cores per plot), thoroughly homogenized into one composite sample per plot, generating 36 composite samples (2 elevations × 2 encroachment stages × 3 months × 3 replicates).

Background soil (R/CK): In unamended control plots, five topsoil cores (0–10 cm) were collected using a five-point cross-sampling method and mixed into a single composite sample per plot (2 elevations × 2 encroachment stages × 3 months × 3 replicates = 36 composite CK samples).

To match the phenological monitoring period, soil data from corresponding growing season months were selected for integrated analyses. Soil samples without additional litter addition were used to represent background soil habitat conditions and to avoid confounding effects of experimental litter addition.

After removing surface litter and visible roots, topsoil samples were collected from each plot. Fresh soil was transported to the laboratory on ice and divided into subsamples. One sample was stored at low temperature for inorganic nitrogen and enzyme activity analyses, one was air-dried for physicochemical measurements, and one was stored at −80 °C for bacterial DNA extraction.

These variables were used to characterize soil nutrient pools, inorganic nitrogen availability, microbial-mediated nutrient transformation potential (urease, alkaline phosphatase, and sucrase), and soil microbial oxidative stress response and aerobic metabolic activity (catalase).

Soil pH: Measured potentiometrically in a 1:5 (*w*/*v*) soil-to-deionized water suspension (2.000 g soil in 10.00 mL water, equilibrated for 30 min, centrifuged at 5000 r/min for 5 min) using a calibrated digital pH meter. Soil Organic Carbon (SOC): Determined by potassium dichromate oxidation spectrophotometry. Soil (0.2000 g) was digested with 0.1000 g HgSO_4_, 5.00 mL of 0.8 mol/L 1/6 K_2_Cr_2_O_7_, and 7.50 mL concentrated H_2_SO_4_ at 135 °C for 30 min on a digestion block. After cooling, the solution was diluted to 100.00 mL, and the supernatant was centrifuged (2000 r/min, 10 min) and measured at 585 nm against a glucose calibration standard curve. Total Nitrogen (TN): Quantified using the Kjeldahl digestion method. Soil (0.5000 g) was digested with 2.0000 g catalyst (K_2_SO_4_:CuSO_4_:Se = 100:10:1) and 5.00 mL concentrated H_2_SO_4_ (200 °C for 20 min, followed by 375 °C for 75 min). Digests were filtered, diluted to 100.00 mL, and an aliquot (10.00 mL) was analyzed on an automated Kjeldahl analyzer (K1306, Shanghai Sonnen Automated Analytical Instrument Co., Ltd., Shanghai, China). Ammonium Nitrogen (NH_4_-N): Extracted by shaking 2.0000 g soil with 10.00 mL of 2 mol/L KCl at 280 r/min for 1 h. Supernatant (5.00 mL) was reacted with 5.00 mL phenol solution and 5.00 mL alkaline sodium hypochlorite, supplemented with 1.00 mL masking agent, diluted to 50.00 mL, and read at 625 nm via indophenol blue colorimetry. Nitrate Nitrogen (NO_3_-N): Extracted by shaking 2.0000 g soil with 10.00 mL of 1 mol/L KCl at 240 r/min for 1 h. Supernatant (5.00 mL) was diluted to 50.00 mL with 1 mol/L KCl and measured via dual-wavelength UV spectrophotometry (SP-1900UV, Shanghai Spectrum Instruments Co., Ltd., Shanghai, China) at 220 nm and 275 nm.

Catalase (CAT): Assayed by reacting 2.0000 g soil with 0.20 mL toluene and 10.00 mL of 0.3% H_2_O_2_ for 20 min on a shaker. Reaction was stopped with 5.00 mL of 3 mol/L H_2_SO_4_, and centrifuged supernatant (10.00 mL) was titrated with 0.1 mol/L KMnO_4_. CAT activity is expressed as mL 0.1 mol/L KMnO_4_ per g dry soil per 20 min and interpreted as an indicator of microbial oxidative stress and aerobic respiration.

Urease (URE): Assayed by incubating 2.0000 g soil with 1.00 mL toluene, 4.00 mL of 10% urea, and 8.00 mL citrate buffer (pH 6.7) at 37 °C for 24 h. Released NH_4_-N in 1.00 mL supernatant was quantified at 578 nm using sodium phenate and sodium hypochlorite, expressed as mg NH_4_-N per g dry soil per 24 h.

Alkaline Phosphatase (ALP): Assayed by incubating 2.0000 g soil with 0.20 mL toluene and 8.00 mL of 0.5% disodium phenyl phosphate at 37 °C for 24 h. Liberated phenol in 5.00 mL filtrate was reacted with 5.00 mL borate buffer (pH 9.0) and 50 µL BQC, and measured at 660 nm, expressed as mg phenol per g dry soil per 24 h.

Sucrase: Assayed by incubating 2.0000 g soil with 7.20 mL of 8% sucrose, 2.00 mL phosphate buffer (pH 5.5), and 0.20 mL toluene at 37 °C for 24 h. Produced reducing sugar in 1.00 mL filtrate was reacted with 3.00 mL DNS reagent in a boiling water bath for 5 min and read at 508 nm, expressed as mg glucose per g dry soil per 24 h.

### 4.6. Soil Bacterial 16S rRNA Sequencing

Total soil microbial genomic DNA was extracted from frozen soil samples and subjected to rigorous quality and concentration verification. Qualified DNA extracts served as templates for PCR amplification targeting the hypervariable V3–V4 region of the bacterial 16S rRNA gene, utilizing sample-specific barcoded primers 338F (5′-ACTCCTACGGGAGGCAGCA-3′) and 806R (5′-GGACTACHVGGGTWTCTAAT-3′). Purified amplicons were quantified, library-constructed, and subjected to high-throughput paired-end sequencing on the Illumina NovaSeq platform (Personal Biotechnology Co., Ltd., Shanghai, China). Raw sequencing reads were demultiplexed and processed through a standardized quality control pipeline. Primer and barcode sequences were trimmed, and raw reads underwent quality filtering, denoising, paired-end merging, and chimera removal to generate non-chimeric Amplicon Sequence Variants (ASVs). High-quality representative ASVs were taxonomically annotated against the SILVA reference database (release 138). To eliminate spurious diversity artifacts caused by variation in sequencing depth across samples, the ASV feature abundance matrix was rarefied to an even sequencing depth across all samples. Raw 16S rRNA gene sequencing data have been deposited in the NCBI Sequence Read Archive (SRA) under BioProject ID PRJNA1484777.

### 4.7. Statistical Analysis

All analyses were conducted in R version 4.5.3. Statistical tests were two-sided, and exact *p*-values were reported wherever available. Effect sizes and 95% confidence intervals were reported where applicable. S and L denote slight and large encroachment, respectively.

For the plant community survey, the observational unit was the plot (*n* = 12; three plots per elevation × encroachment-stage habitat). Species weighted cover within each plot was calculated as abundance × single-plant cover, and relative cover was calculated as the weighted cover of a species divided by the summed weighted cover of all species in that plot × 100%. Bray–Curtis dissimilarities were calculated from the plot-level weighted species cover matrix and visualized using principal coordinates analysis. Differences among the four habitats were tested by PERMANOVA with 9999 permutations, and homogeneity of multivariate dispersion was assessed using PERMDISP. The relative cover of D. angustifolia, species richness, Shannon diversity, and Pielou evenness were analyzed using two-way ANOVA with elevation, encroachment stage, and their interaction as predictors.

For the growth trait analysis, the observational unit was the marked individual. Ten individuals were repeatedly measured in each of four selected habitats. Inferential repeated-measures analyses used the six dates for which individual-level measurements were available in all four habitats. Plant height, leaf length, leaf width, and stem diameter were analyzed using Gaussian linear mixed effects models; leaf number and tiller number were analyzed using Poisson generalized linear mixed effects models. Elevation, encroachment stage, census date, and their interactions were specified as fixed effects, with individual identity fitted as a random intercept. Final census and seasonal maximum traits were summarized using means and standard errors and analyzed using two-way ANOVA. Within each elevation, L − S standardized mean differences were expressed as Hedges’ g with 95% confidence intervals. Because each elevation × encroachment combination was represented by one selected habitat, inference from these analyses was restricted to differences among the selected habitats and did not establish replicated habitat-level treatment effects.

Litter mass remaining was summarized by elevation, encroachment stage, and retrieval month. S and L habitats were compared within each elevation and month using Welch’s *t*-tests. Decomposition rate constants were evaluated using linear models containing elevation, encroachment stage, and their interaction. Soil physicochemical properties and enzyme activities were analyzed using factorial models with elevation, encroachment stage, and month as explanatory variables. Catalase was interpreted separately as an oxidative process indicator rather than as an enzyme directly representing nutrient transformation potential.

Bacterial alpha-diversity indices were compared between encroachment stages within each elevation × month combination using Welch’s *t*-tests. Bacterial community composition was analyzed using Bray–Curtis dissimilarity, factorial PERMANOVA, and PERMDISP. Integrated heatmaps and correlation analyses were used to explore habitat-level associations among plant traits, litter decomposition, soil properties, enzyme activities and bacterial community characteristics. Figures were produced using R packages including ggplot2, dplyr, tidyr, vegan, broom, patchwork and related visualization packages. Statistical significance was assessed at *p* < 0.05 unless otherwise stated.

## 5. Conclusions

*D. angustifolia* encroachment in the Changbaishan alpine tundra was associated with marked differences in abundance-weighted plant community composition, plant growth trait profiles, litter decomposition, soil properties, enzyme activities, and bacterial community composition. The selected growth trait habitats showed elevation-dependent differences in plant height, leaf dimensions, stem diameter, and tiller production, while litter decomposition and belowground variables also differed between spatial habitat states. This reframes the ecological significance of *D. angustifolia* expansion from a simple rise in dominant species cover to a broader habitat reorganization and suggests that assessing alpine tundra vegetation change under climate warming should weigh not only an encroaching species establishment capacity but also its local space occupancy strategy and its litter decomposition dynamics effects on soil habitats.

## Data Availability

The raw soil bacterial 16S rRNA gene sequencing data generated in this study have been deposited in the NCBI Sequence Read Archive (SRA) repository under BioProject accession number PRJNA1484777. All other relevant data including plant traits, soil physicochemical properties, enzyme activities, and litter decomposition rates supporting the findings of this study are available within the article or from the corresponding author upon reasonable request.

## Supplementary Materials

The following supporting information can be downloaded at: https://www.mdpi.com/article/10.3390/plants15182776/s1.

## Abbreviations

The following abbreviations are used in this manuscript:

C:N ratio	Carbon-to-Nitrogen Ratio
SOC	Soil Organic Carbon
TN	Total Nitrogen
S	Slightly Encroached (Habitat/Plot)
L	Largely Encroached (Habitat/Plot)
